# Supplementary material for: Structural dynamics of the CROPs domain control stability and toxicity of Paeniclostridium sordellii lethal toxin
Source: Nat Commun. 2023 Dec 19;14:8426. doi: 10.1038/s41467-023-44169-z (PMC10730571; doi:10.1038/s41467-023-44169-z)
Supplement: Supplementary file 1 — Supplementary Information [file 41467_2023_44169_MOESM1_ESM.pdf]

Supplementary Information

Structural dynamics of the CROPs domain control stability and toxicity of *Paeniclostridium sordellii* lethal toxin

Yao Zhou[1,2,3,4,#], Xiechao Zhan[1,2,3,#,*], Jianhua Luo[1,2,3,4], Diyin Li[1,2,3,4], Ruoyu Zhou[1,2,3], Jiahao Zhang[1,2,3], Zhenrui Pan[1,2,3], Yuanyuan Zhang[1,2,3], Tianhui Jia[1,2,3], Xiaofeng Zhang[1,2,3], Yanyan Li[1,2,3], and Liang Tao[1,2,3,4,*]

[1]Center for Infectious Disease Research, Westlake Laboratory of Life Sciences and Biomedicine, Hangzhou, Zhejiang 310024, China

[2]Key Laboratory of Structural Biology of Zhejiang Province, School of Life Sciences, Westlake University, Hangzhou, Zhejiang 310024, China

[3]Westlake Institute for Advanced Study, Hangzhou, Zhejiang 310024, China

[4]Research Center for Industries of the Future, Westlake University, Hangzhou, Zhejiang 310024, China

[#]These authors contributed equally to this work.

*Corresponding to:

Xiechao Zhan, Ph.D. Email: zhanxiechao@westlake.edu.cn

Liang Tao, Ph.D.   Email: taoliang@westlake.edu.cn

Supplementary Figures

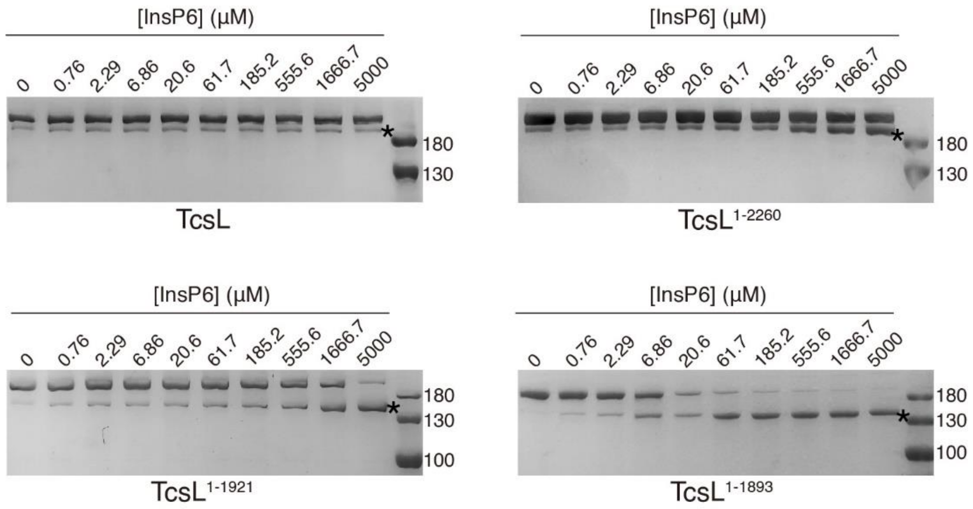

Supplementary Fig. 1 │TcsL variants with the CROPs partly deleted show enhanced InsP6-induced autoproteolysis. The autocleavage of TcsL, TcsL$^{1\text{-}2260}$, TcsL$^{1\text{-}1921}$, and TcsL$^{1\text{-}1893}$ induced by gradient concentrations of InsP6 was shown by Coomassie-stained SDS-PAGE gels. The asterisks indicate the cleavaged fragments.

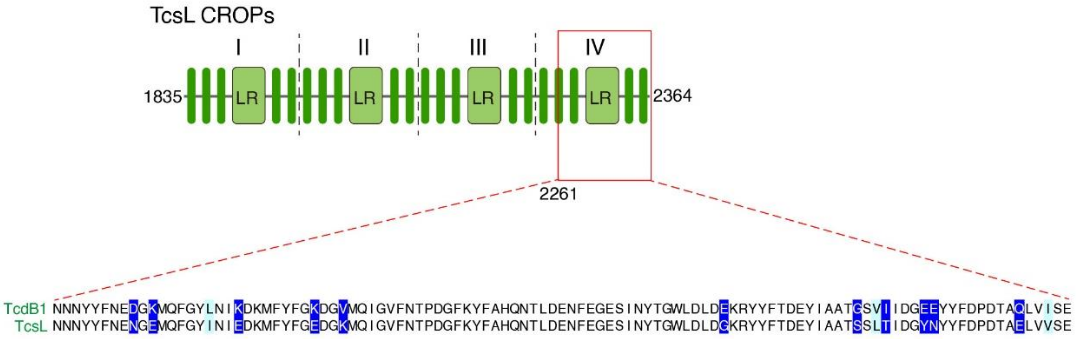

Supplementary Fig. 2 │ A Schematic drawing of TcsL CROPs. The last 104 amino acids (residues 2261 to 2364) of TcsL are compared with the homologous sequence from TcdB1.

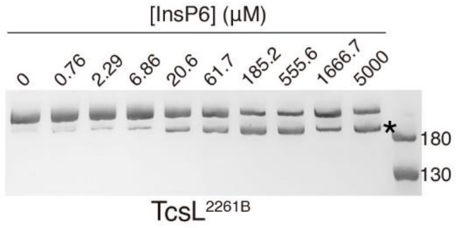

Supplementary Fig. 3 │ TcsL^{2261B} is vulnerable to InsP6-induced autoproteolysis. The autocleavage of TcsL^{2261B} induced by gradient concentrations of InsP6 was shown by Coomassie-stained SDS-PAGE gels. The asterisks indicate the cleavaged fragments.

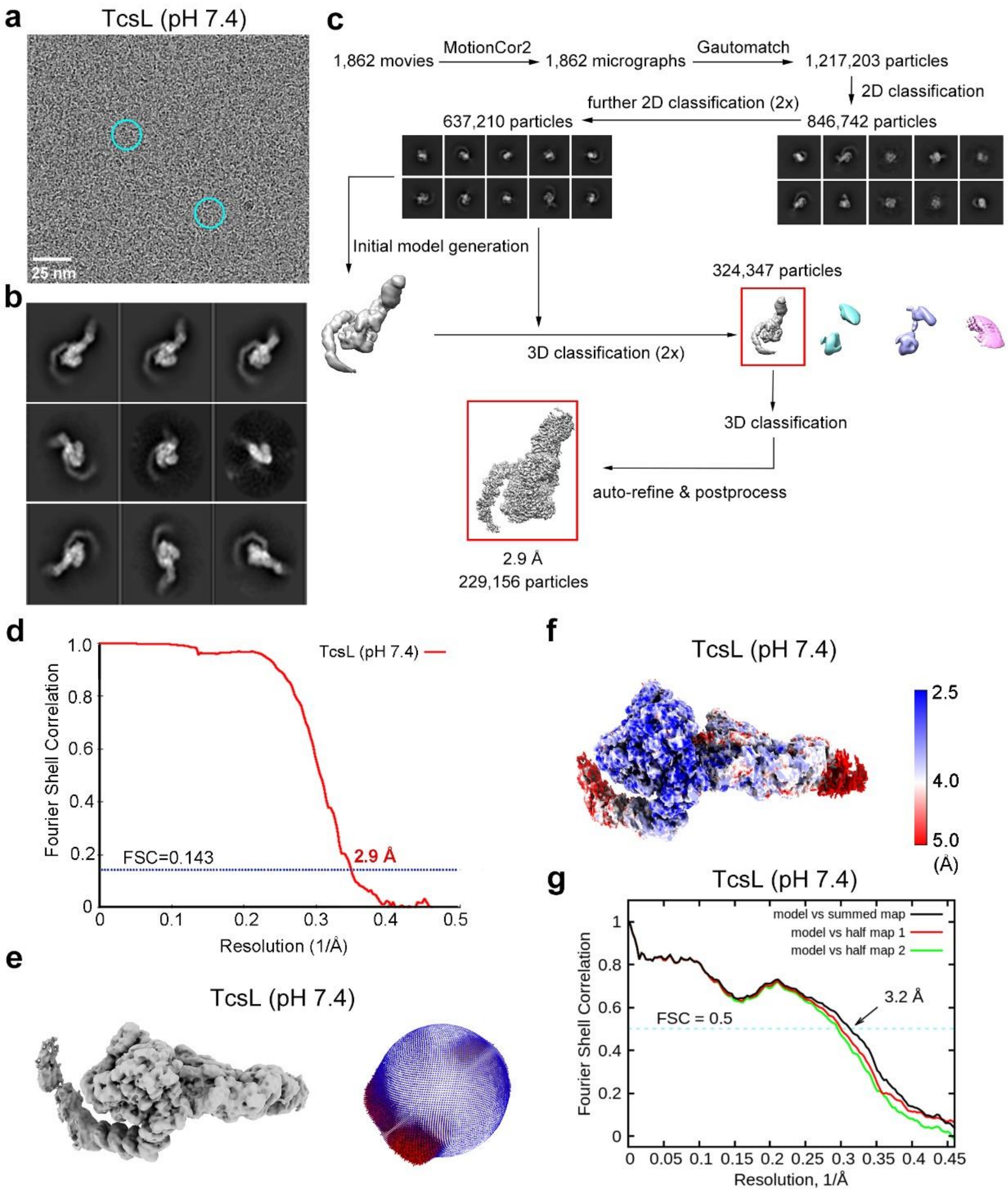

Supplementary Fig. 4 │ Cryo-EM reconstruction and analysis of TcsL at pH 7.4. (a) A representative EM micrograph of the TcsL at pH 7.4. Scale bar, 25 nm. (b) Representative 2D class averages of the TcsL at pH 7.4. (c) A flow chart of cryo-EM data processing for the TcsL at pH 7.4. (d) The FSC curves for the final refinement of TcsL at pH 7.4. (e) The angular distribution of the particles used for the final reconstruction of the TcsL at pH 7.4. (f) The local resolutions with color-coded for different regions of the TcsL at pH 7.4. (g) The FSC curves for cross-validation between the models and the cryo-EM maps for the TcsL at pH 7.4. Shown here are the FSC curves between the final refined atomic model and the reconstruction from all particles (black), between the model refined in the reconstruction from only half of the particles and the reconstruction from that same half (red), and between that same model and the reconstruction from the other half of the particles (green).

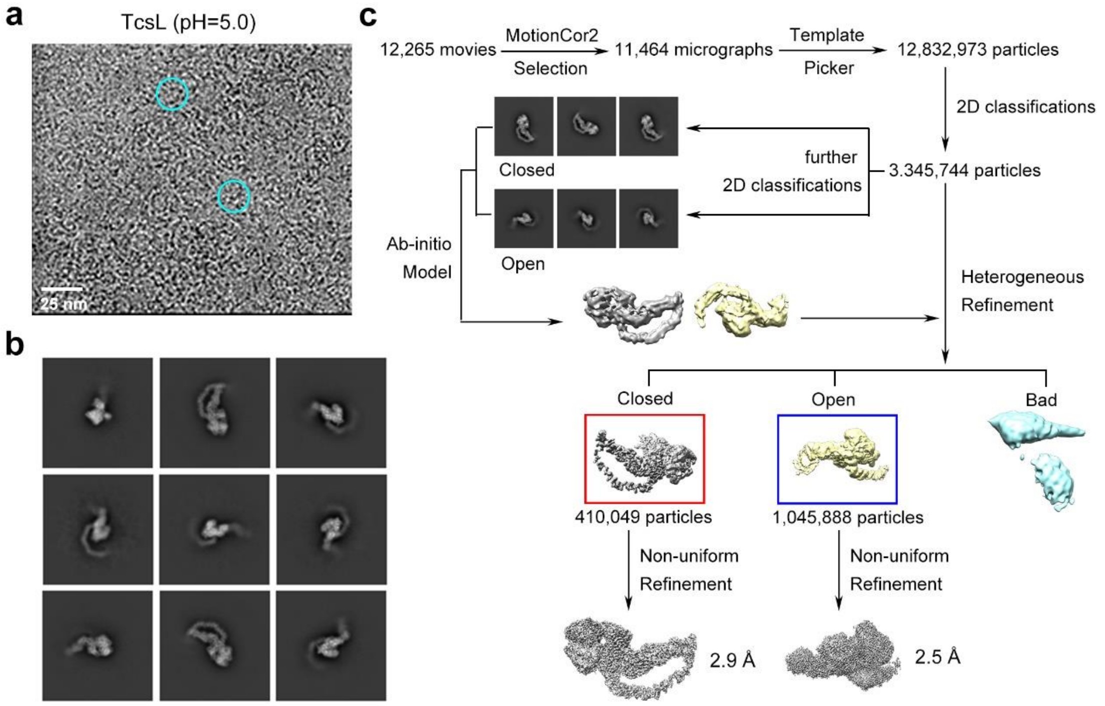

Supplementary Fig. 5 | Cryo-EM reconstruction of the TcsL at pH 5.0. (a) A representative EM micrograph of the TcsL at pH 5.0. Scale bar, 25 nm. (b) Representative 2D class averages of the TcsL at pH 5.0. (c) A flow chart of cryo-EM data processing for the TcsL at pH 5.0.

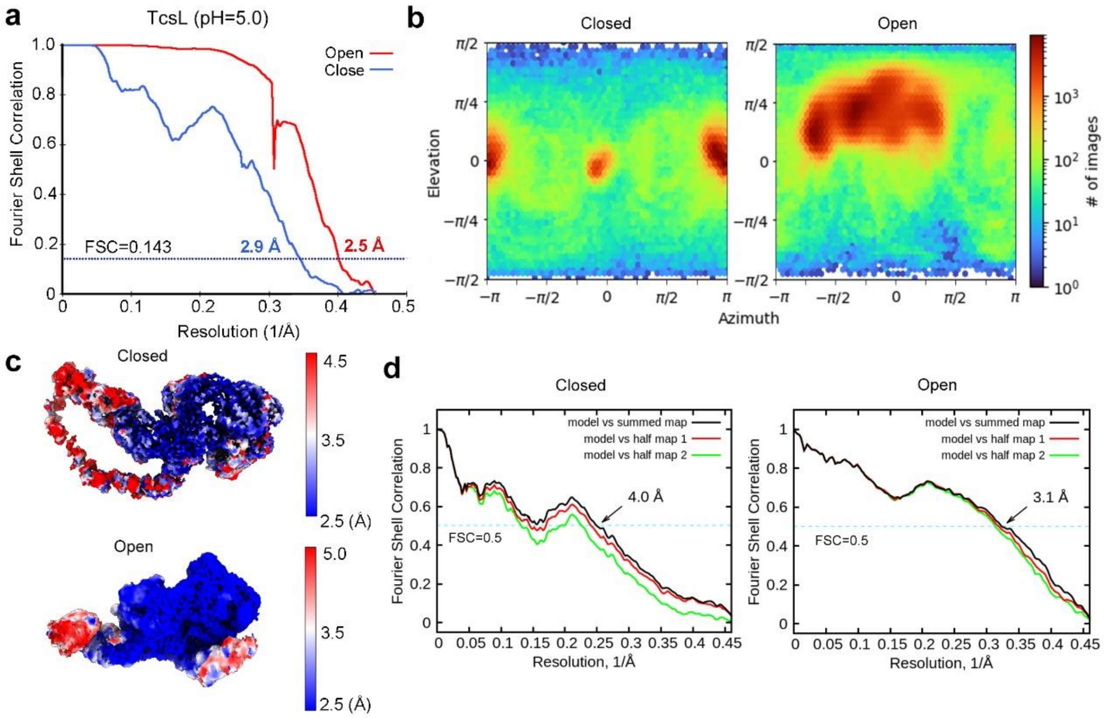

Supplementary Fig. 6 │ Cryo-EM analysis of TcsL at pH 5.0. (a) The FSC curves for the final refinement of TcsL at pH 5.0. (b) The angular distribution of the particles used for the final reconstruction of the TcsL at pH 5.0. (c) The local resolutions with color-coded for different regions of the TcsL at pH 5.0. (d) The FSC curves for cross-validation between the models and the cryo-EM maps for the TcsL at pH 5.0.

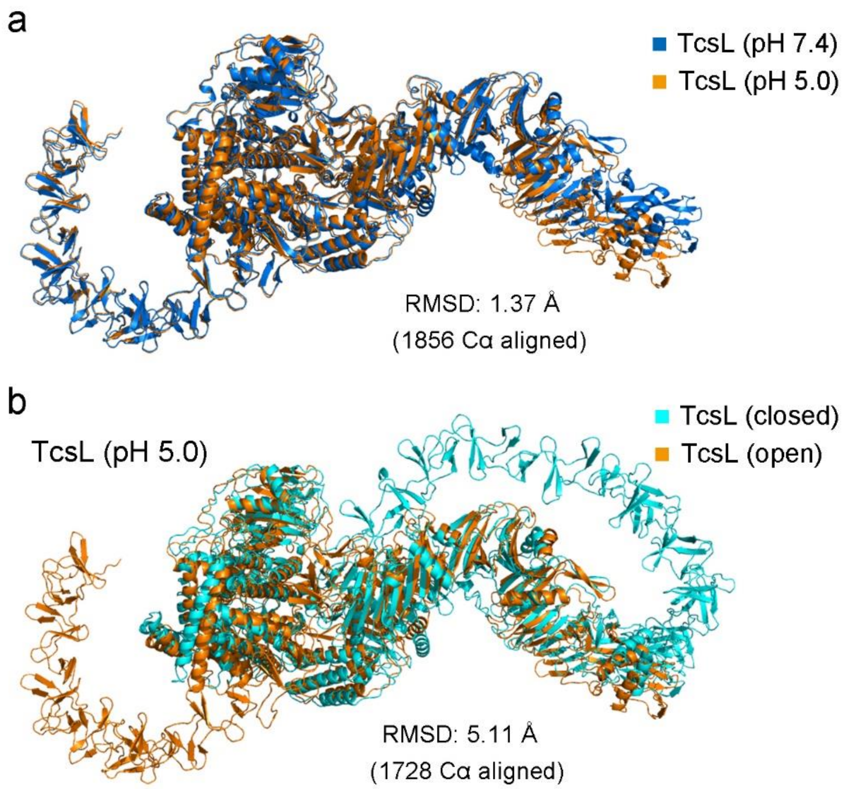

Supplementary Fig. 7 │Low-pH induces conformation change of TcsL. (a) Superimposed structures of the TcsL at neutral pH and at the acidic pH in its open conformation. (b) Superimposed structures of the TcsL at the acidic pH in its open and closed conformations.

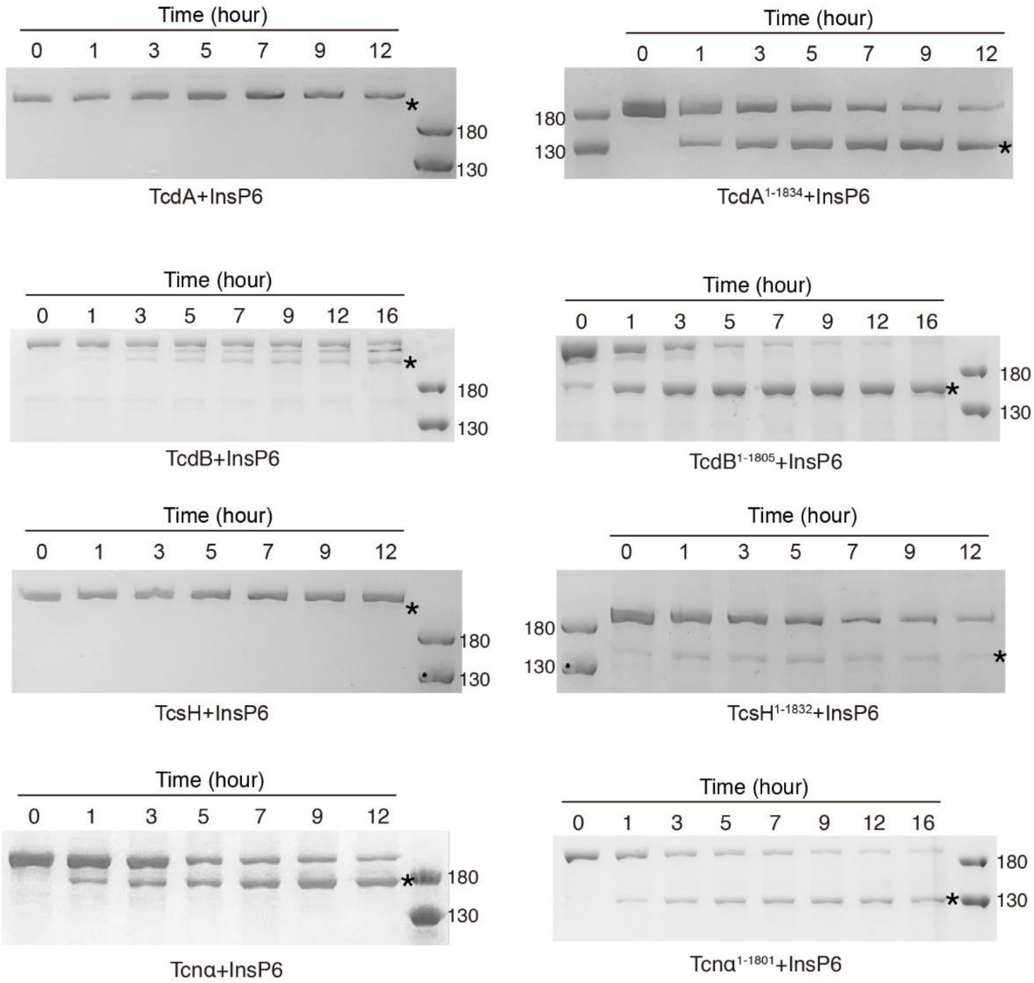

Supplementary Fig. 8 │ The CROP-less LCTs show increased autoproteolysis compared to the full-length toxins. The autocleavage of TcdA, TcdA$^{1\text{-}1834}$, TcdB, TcdB$^{1\text{-}1805}$, TcsH, TcsH$^{1\text{-}1832}$, Tcnα, and Tcnα$^{1\text{-}1801}$ in the presence of 100 μM InsP6 was shown by Coomassie-stained SDS-PAGE gels. The asterisks indicate the cleavaged fragments.

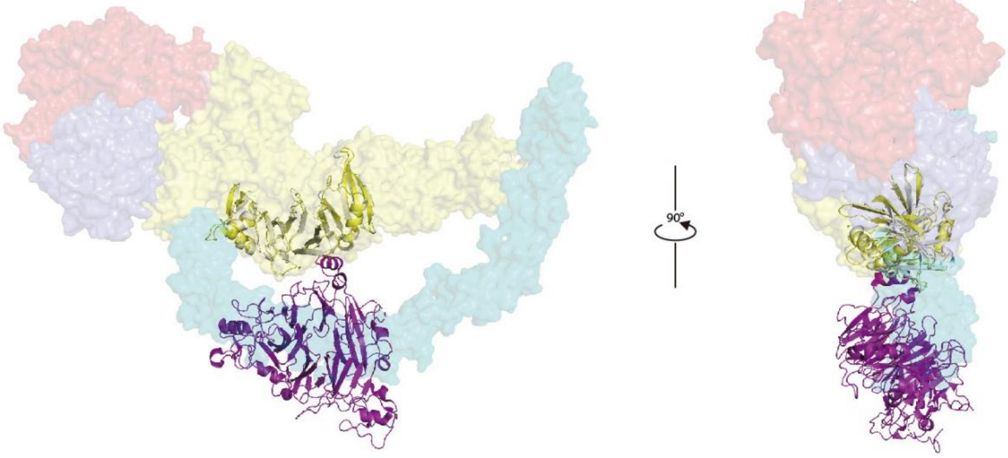

Supplementary Fig. 9 │ The CROPs domain of TcsL may hinder the SEMA6A binding. Co-structure of the TcsL$^{1285\text{-}1804}$-SEMA6A$^{19\text{-}570}$ complex (PDB 6WTS) is superimposed to the full-length TcsL structure in the closed conformation.

Supplementary Table

Supplementary Table S1 | Statistics of the cryo-EM structures in this study.

	TcsL (pH 7.4)	TcsL (pH 5.0)	
		Closed	Open
**Data collection**			
EM equipment		FEI Titan Krios	
Voltage (kV)		300	
Detector		K3	
Magnification		81,000	
Pixel size (Å)		1.087	
Electron dose (e-/Å^2)		50	
Defocus range (μm)		1.5~2.0	
**Reconstruction**			
Software	RELION3.0	cryoSPARC	
Symmetry		C1	
Number of particles	229,156	410,049	1,045,888
Final masked resolution (Å)	2.9	2.9	2.5
Map sharpening B-factor (Å^2)	-90.5	-86.6	-99.6
EMDB code	EMD-36141	EMD-38010	EMD-38011
**Model building**			
Software		Coot-0.8.9/Chimera	
Refinement		Phenix	
Protein residues	2,355	2,363	2,303
B factors (Å^2)	64.7	42.0	36.8
PDB code	8JB5	8X2H	8X2I
**Validation**			
R.m.s deviations			
Bonds length (Å)	0.008	0.004	0.009
Bonds Angle (°)	0.882	0.757	0.924
Ramachandran plot statistics (%)			
Favored	94.00	91.11	94.25
Outlier	0.26	0.17	0.17
Clashscore	13.77	15.61	13.82
CaBLAM outliers (%)	1.9	3.5	1.9
MolProbity score	2.66	3.07	2.77
