# Supplementary material for: Structural dynamics of the CROPs domain control stability and toxicity of Paeniclostridium sordellii lethal toxin
Source: Nat Commun. 2023 Dec 19;14:8426. doi: 10.1038/s41467-023-44169-z (PMC10730571; doi:10.1038/s41467-023-44169-z)
Supplement: Supplementary file 3 — Description of Additional Supplementary Files [file 41467_2023_44169_MOESM3_ESM.pdf]

<u>Description of Additional Supplementary Files</u>

Supplementary Movie 1 : Conformational change of TcsLfrom open to closed state.
