# Supplementary material for: Structural dynamics of the CROPs domain control stability and toxicity of Paeniclostridium sordellii lethal toxin
Source: Nat Commun. 2023 Dec 19;14:8426. doi: 10.1038/s41467-023-44169-z (PMC10730571; doi:10.1038/s41467-023-44169-z)
Supplement: Supplementary file 5 — Reporting Summary [file 41467_2023_44169_MOESM5_ESM.pdf]

nature portfolio

Corresponding author(s): Xiechao Zhan
Liang Tao

Last updated by author(s): Nov 24, 2023

Reporting Summary

Nature Portfolio wishes to improve the reproducibility of the work that we publish. This form provides structure for consistency and transparency in reporting. For further information on Nature Portfolio policies, see our Editorial Policies and the Editorial Policy Checklist.

Statistics

For all statistical analyses, confirm that the following items are present in the figure legend, table legend, main text, or Methods section.

n/a	Confirmed	
☐	☒	The exact sample size (*n*) for each experimental group/condition, given as a discrete number and unit of measurement
☐	☒	A statement on whether measurements were taken from distinct samples or whether the same sample was measured repeatedly
☐	☒	The statistical test(s) used AND whether they are one- or two-sided<br>*Only common tests should be described solely by name; describe more complex techniques in the Methods section.*
☒	☐	A description of all covariates tested
☒	☐	A description of any assumptions or corrections, such as tests of normality and adjustment for multiple comparisons
☐	☒	A full description of the statistical parameters including central tendency (e.g. means) or other basic estimates (e.g. regression coefficient) AND variation (e.g. standard deviation) or associated estimates of uncertainty (e.g. confidence intervals)
☐	☒	For null hypothesis testing, the test statistic (e.g. *F*, *t*, *r*) with confidence intervals, effect sizes, degrees of freedom and *P* value noted<br>*Give P values as exact values whenever suitable.*
☒	☐	For Bayesian analysis, information on the choice of priors and Markov chain Monte Carlo settings
☒	☐	For hierarchical and complex designs, identification of the appropriate level for tests and full reporting of outcomes
☒	☐	Estimates of effect sizes (e.g. Cohen's *d*, Pearson's *r*), indicating how they were calculated

Our web collection on statistics for biologists contains articles on many of the points above.

Software and code

Policy information about availability of computer code

Data collection	Olympus CellsSens Standard 2.1<br>Thermo Fisher Scientific EPU
Data analysis	CHIMERA (http://www.cgl.ucsf.edu/chimera)<br>COOT (https://www2.mrc-lmb.cam.ac.uk/personal/pemsley/coot/)<br>Gctf (https://en.wikibooks.org/w/index.php?title=Software_Tools_For_Molecular_Microscopy&stable=0#Gctf)<br>RELION3.0 (https://www2.mrc-lmb.cam.ac.uk/relion)<br>MotionCor2 (https://msg.ucsf.edu/em/software/motioncor2.html)<br>Gautomatch (https://www2.mrc-lmb.cam.ac.uk/research/locally-developed-software/zhang-software/#gauto)<br>OriginPro v8.5 (https://www.originlab.com/)<br>GraphPad Prism v9.3 (https://www.graphpad.com:443/)

For manuscripts utilizing custom algorithms or software that are central to the research but not yet described in published literature, software must be made available to editors and reviewers. We strongly encourage code deposition in a community repository (e.g. GitHub). See the Nature Portfolio guidelines for submitting code & software for further information.

Data

Policy information about availability of data

All manuscripts must include a data availability statement. This statement should provide the following information, where applicable:

- Accession codes, unique identifiers, or web links for publicly available datasets
- A description of any restrictions on data availability
- For clinical datasets or third party data, please ensure that the statement adheres to our policy

The atomic coordinates for the TcsL at pH 7.4 and at the pH 5.0 in its open and closed conformations have been deposited in the Protein Data Bank (PDB) with the accession codes 8JB5, 8X2H, and 8X2I, respectively. The EM maps have been deposited in the EMDB with the accession codes EMD-36141, EMD-38010, and EMD-38011 for the TcsL at pH 7.4 and 5.0 in its open and closed conformations, respectively. The other data that support the findings of this study are available in a publicly accessible repository. Source data are provided with the paper.

Research involving human participants, their data, or biological material

Policy information about studies with human participants or human data. See also policy information about sex, gender (identity/presentation), and sexual orientation and race, ethnicity and racism.

Reporting on sex and gender	N/A
Reporting on race, ethnicity, or other socially relevant groupings	N/A
Population characteristics	N/A
Recruitment	N/A
Ethics oversight	N/A

Note that full information on the approval of the study protocol must also be provided in the manuscript.

Field-specific reporting

Please select the one below that is the best fit for your research. If you are not sure, read the appropriate sections before making your selection.

☒ Life sciences ☐ Behavioural & social sciences ☐ Ecological, evolutionary & environmental sciences

For a reference copy of the document with all sections, see nature.com/documents/nr-reporting-summary-flat.pdf

Life sciences study design

All studies must disclose on these points even when the disclosure is negative.

Sample size	For all cell rounding experiments: n=6 for each group.<br>For pathological assay: n=10 mice per group.<br>For toxin challenge assay: n=5 mice per group.<br>Sample size was determined based on previous study and knowledge. Each sample size was selected so that a reasonable researcher would conclude that the size is sufficient to draw a statistical conclusion. For in vitro studies, at least three biological replicates were performed. For in vivo toxicological studies for large clostridial toxins, 5-10 mice per group is considered sufficient. (Tao et al. Nature 2016; Chen et al. Nat Commun 2021; Luo et al. Cell 2022; etc.)
Data exclusions	No data exclusions.
Replication	All experiments were replicated at least three times. All attempts at replication are successful.
Randomization	Samples were allocated into experimental groups randomly.
Blinding	The stained tissue sections were scored blinded by two pathologists.<br>For other experiments, blinding was not performed as virtually those data are quantitative and would not easily subject to operator bias.

Reporting for specific materials, systems and methods

We require information from authors about some types of materials, experimental systems and methods used in many studies. Here, indicate whether each material, system or method listed is relevant to your study. If you are not sure if a list item applies to your research, read the appropriate section before selecting a response.

Materials & experimental systems

n/a	Involved in the study	
☒	☐	Antibodies
☐	☒	Eukaryotic cell lines
☒	☐	Palaeontology and archaeology
☐	☒	Animals and other organisms
☒	☐	Clinical data
☒	☐	Dual use research of concern
☒	☐	Plants

Methods

n/a	Involved in the study	
☒	☐	ChIP-seq
☒	☐	Flow cytometry
☒	☐	MRI-based neuroimaging

Eukaryotic cell lines

Policy information about cell lines and Sex and Gender in Research

Cell line source(s)	HeLa (H1, CRL-1958) and A549 (CCL-185) cells were originally obtained from ATCC.
Authentication	HeLa and A549 cells were authenticated via STR profiling (Shanghai Biowing Biotechnology Co. LTD, Shanghai, China).
Mycoplasma contamination	These cells were tested negative for mycoplasma contamination.
Commonly misidentified lines (See ICLAC register)	No commonly misidentified lines were used in this study.

Animals and other research organisms

Policy information about studies involving animals; ARRIVE guidelines recommended for reporting animal research, and Sex and Gender in Research

Laboratory animals	BALB/c mice (6-8 weeks, male, specific-pathogen-free) were purchased from Laboratory Animal Resources Center at Westlake University (Hangzhou, China). Mice were housed in specific-pathogen-free micro-isolator cages with free access to drinking water and food during the experiments. All mice had a 12-hour cycle of light/darkness (7am-7pm), housed at 20-24°C with 40-60% of humidity, and monitored under the care of full-time staff.
Wild animals	No wild animals were used in this study.
Reporting on sex	The mice used in this study are all male. Physiological cycles of female animals may affect the stability of toxicological experiments; thus male mice were chosen.
Field-collected samples	No field-collected samples were used in this study.
Ethics oversight	All animal procedures reported herein were performed following the institutional guidelines and approved by the Institutional Animal Care and Use Committee at Westlake University (IACUC Protocol #19-010-TL and #22-018-TL-5).

Note that full information on the approval of the study protocol must also be provided in the manuscript.

Plants

Seed stocks	N/A
Novel plant genotypes	N/A
Authentication	N/A
